# hTERT Promotes CRC Proliferation and Migration by Recruiting YBX1 to Increase NRF2 Expression

**DOI:** 10.3389/fcell.2021.658101

**Published:** 2021-05-17

**Authors:** Chunli Gong, Huan Yang, Sumin Wang, Jiao Liu, Zhibin Li, Yiyang Hu, Yang Chen, Yu Huang, Qiang Luo, Yuyun Wu, En Liu, Yufeng Xiao

**Affiliations:** ^1^Department of Gastroenterology, Xinqiao Hospital, Third Military Medical University, Chongqing, China; ^2^Department of Endoscope, General Hospital of Shenyang Military Region, Shenyang, China

**Keywords:** hTERT, NRF2, colorectal cancer, progression, YBX1

## Abstract

High human telomerase reverse transcriptase (hTERT) expression is related to severe Colorectal Cancer (CRC) progression and negatively related to CRC patient survival. Previous studies have revealed that hTERT can reduce cancer cellular reactive oxygen species (ROS) levels and accelerate cancer progression; however, the mechanism remains poorly understood. NFE2-related factor 2 (NRF2) is a molecule that plays a significant role in regulating cellular ROS homeostasis, but whether there is a correlation between hTERT and NRF2 remains unclear. Here, we showed that hTERT increases CRC proliferation and migration by inducing NRF2 upregulation. We further found that hTERT increases NRF2 expression at both the mRNA and protein levels. Our data also revealed that hTERT primarily upregulates NRF2 by increasing NRF2 promoter activity rather than by regulating NRF2 mRNA or protein stability. Using DNA pull-down/MS analysis, we found that hTERT can recruit YBX1 to upregulate NRF2 promoter activity. We also found that hTERT/YBX1 may localize to the P2 region of the NRF2 promoter. Taken together, our results demonstrate that hTERT facilitates CRC proliferation and migration by upregulating NRF2 expression through the recruitment of the transcription factor YBX1 to activate the NRF2 promoter. These results provide a new theoretical basis for CRC treatment.

## Introduction

Colorectal cancer (CRC) is the third most frequently occurring carcinoma and the second most common cause of cancer-related death in the world (Rahman et al., 2019; Cui et al., 2020). The main cause of CRC patient death may be the high probability of invasion and metastasis of CRC cells. Almost 30% of patients who are diagnosed with early−stage CRC will develop metastatic disease (Yin et al., 2019). It is paramount that additional prognostic CRC biomarkers and treatment targets be identified based on a better understanding of the mechanisms underlying CRC metastasis.

Human telomerase reverse transcriptase (hTERT) is an important component of human telomerase that synthesizes telomeric DNA to maintain and increase telomere length, ultimately leading to cellular immortality (Li et al., 2017). hTERT is only expressed in immortalized cells and most tumor cells but not in normal somatic cells (Liu et al., 2006). hTERT is expressed in many cancer types (including CRC) and plays a crucial role in oncogenesis by providing proliferation, survival and anti-apoptotic signals necessary for tumor progression (Sirera et al., 2008; Loyon et al., 2019). hTERT can regulate CRC via multiple pathways; for example, hTERT can be upregulated at the mRNA and protein level by KRT23 to promote CRC growth (Zhang N. et al., 2017), and CRC proliferation can be increased through the circFMN2/miR-1182/hTERT axis (Li et al., 1979). hTERT can also recruit ZEB1 to act on the promoter of E- cadherin to increase CRC metastasis (Qin et al., 2016). Moreover, hTERT can facilitate tumor metastasis by upregulating ITGB1 (Hu et al., 2017). Previous studies have found that hTERT overexpression reduces intracellular basal ROS levels and inhibits endogenous ROS production in response to stimuli that induce cellular ROS generation, thus decreasing ROS-mediated apoptosis (Indran et al., 2011; Dilshara et al., 2019); However, the mechanisms remain unclear.

NFE2-related factor 2 (NRF2) is a transcription factor that principally maintains the cellular ROS balance and plays dual roles in cancer proliferation, invasion and cell differentiation (Sporn and Liby, 2012; Kahroba et al., 2019; Pölönen et al., 2019). NRF2 stability is tightly controlled by Kelch-like ECH-associated protein 1 (KEAP1) in many cancer types (Sanghvi et al., 2019). Under normal physiological conditions, NRF2 is anchored by KEAP1, but in the presence of ROS, NRF2 dissociates from KEAP1 and interacts with its target genes that contain ARE (antioxidant response element) sequences and thus regulates cellular redox homeostasis, protecting cells from ROS damage (Liu C. et al., 2020). According to TCGA, NRF2 is highly expressed in CRC patients, and our previous study found that both hTERT and NRF2 were highly expressed and had a positive relationship in CRC, but the mechanism of hTERT-NRF2 regulation is unclear.

In the present study, we aimed to investigate the mechanism by which hTERT regulates NRF2 to enhance the proliferation and migration of CRC cells. We demonstrate that hTERT can act as a co-activator to recruit the transcriptional factor YBX1 to the promoter region of NRF2 to increase its expression, ultimately promoting proliferation and migration of CRC cells. Our findings provide novel insights into the crucial role of hTERT in the progression of CRC and may provide a new theoretical basis for the prevention and treatment of CRC.

## Materials and Methods

### Cell Lines

The colon cancer cell lines RKO, HCT116, and sw620 were obtained from the Chinese Academy of Sciences (Shanghai, China) and cultivated in high-glucose DMEM (HyClone, Waltham, MA, United States) with 100 units/ml penicillin, 100 g/ml streptomycin, and 10% FBS at 37°C in an atmosphere of 5% CO_2_.

### RNA Extraction, Reverse Transcription, and Quantitative Real-Time Polymerase Chain Reaction (qRT-PCR)

Total RNA was extracted from the frozen tissues and cell lines using RNAiso Plus reagent (TaKaRa, Dalian, China) according to the manufacturer’s protocol. Reverse transcription was performed using PrimeScript RT Master Mix (TaKaRa, Dalian, China) according to the manufacturer’s instructions. Then, the expression of target genes was determined with a SYBR Premix Ex Taq II Kit (TaKaRa, Dalian, China) and a Step OnePlus system (Applied Biosystems, Forster City, CA, United States). The experimental settings were as follows: hold 95°C 10 min; cycling (95°C for 30 s; 56°C for 30 s; 72°C for 30 s with fluorescence measurement for 40 cycles). The 2^−ΔΔCt^ method was applied to detect fold changes. The primer sequences used for quantitative real-time polymerase chain reaction (qRT-PCR) are listed in Table 1. GAPDH gene expression was used as a reference. All reactions were repeated in triplicate.

**TABLE 1:** Primer sequences used in this paper.

Primers	Sequences	Application	Company
hTERT-F	5′ GCCGATTGTGAACATGGACTACG 3′	qRT-PCR	Sangon Biotech, Shanghai, China
hTERT-R	5′ GCTCGTAGTTGAGCACGCTGAA 3′	qRT-PCR	Sangon Biotech, Shanghai, China
NRF2-F	5′ CGCTTGGAGGCTCATCTCAC 3′	qRT-PCR	Sangon Biotech, Shanghai, China
NRF2-R	5′ TGCAATTCTGAGCAGCCACT 3′	qRT-PCR	Sangon Biotech, Shanghai, China
GAPDH-F	5′ GTCTCCTCTGACTTCAACAGCG 3′	qRT-PCR	Sangon Biotech, Shanghai, China
GAPDH-R	5′ ACCACCCTGTTGCTGTAGCCAA 3′	qRT-PCR	Sangon Biotech, Shanghai, China
NRF2 promoter-F	5′ TTCTGCCGGTCTTGCTTACAGT 3′	PCR	Sangon Biotech, Shanghai, China
NRF2 promoter-R	5′ GGAGTTGCAGAACCTTGCCC 3′	PCR	Sangon Biotech, Shanghai, China
ILF3-F	5′ GATGGTTCTGGCATTTATGACC 3′	qRT-PCR	Sangon Biotech, Shanghai, China
ILF3-R	5′ CTCTGTGTGATATCTTCCCGTT 3′	qRT-PCR	Sangon Biotech, Shanghai, China
XRCC5-F	5′ GTGCGGTCGGGGAATAAGG 3′	qRT-PCR	Sangon Biotech, Shanghai, China
XRCC5-R	5′ GGGGATTCTATACCAGGAATGGA 3′	qRT-PCR	Sangon Biotech, Shanghai, China
YBX1-F	5′ GGGGACAAGAAGGTCATCGC 3′	qRT-PCR	Sangon Biotech, Shanghai, China
YBX1-R	5′ CGAAGGTACTTCCTGGGGTTA 3′	qRT-PCR	Sangon Biotech, Shanghai, China
P1-F	5′ TTGGCAGATTGGAGCACAAAGGAG 3′	ChIP-qPCR	Sangon Biotech, Shanghai, China
P1-R	5′ AGCCTGGCGACAGAGTGAGAC 3′	ChIP-qPCR	Sangon Biotech, Shanghai, China
P2-F	5′ ACTGCAACCTCCGCCTCCTG 3′	ChIP-qPCR	Sangon Biotech, Shanghai, China
P2-R	5′ CCAACGTGGTGAAACCCTGTCTC 3′	ChIP-qPCR	Sangon Biotech, Shanghai, China
P3-F	5′ GGGCAAAGCAAGGGCTCAGG 3′	ChIP-qPCR	Sangon Biotech, Shanghai, China
P3-R	5′ TCTCAAGACCACCCACGTCAAGG 3′	ChIP-qPCR	Sangon Biotech, Shanghai, China
P4-F	5′ ATCCTGGGAGTGTCAAATTATGCA 3′	ChIP-qPCR	Sangon Biotech, Shanghai, China
P4-R	5′ AACCACACACACACCCCTGA 3′	ChIP-qPCR	Sangon Biotech, Shanghai, China
P5-F	5′ ACTGACCACTCTCCGACCTAAAGG 3′	ChIP-qPCR	Sangon Biotech, Shanghai, China
P5-R	5′ TGAACGCCCTCCTCTGAACTCC 3′	ChIP-qPCR	Sangon Biotech, Shanghai, China
P2-1-F	5′ CCTCCTGGGTTCAAGCAATTCTCC 3′	ChIP-qPCR	Sangon Biotech, Shanghai, China
P2-2-R	5′ CAACGTGGTGAAACCCTGTCTCTAC 3′	ChIP-qPCR	Sangon Biotech, Shanghai, China
P2-2-F	5′CCAAAGTGCTGGGATTATAGGCGTTA3′	ChIP-qPCR	Sangon Biotech, Shanghai, China
P2-2-R	5′TTGTGATACCTTGCTCCAGATTGCTC3′	ChIP-qPCR	Sangon Biotech, Shanghai, China
P2-3-F	5′ ATGAGCAATCTGGAGCAAGGTATCAC3′	ChIP-qPCR	Sangon Biotech, Shanghai, China
P2-3-R	5′ CCTGAATCATTTGCTGTCTTTGGGAA3′	ChIP-qPCR	Sangon Biotech, Shanghai, China
P2-4-F	5′ GAAGGCCGTCTTCCCAAAGA 3′	ChIP-qPCR	Sangon Biotech, Shanghai, China
P2-4-R	5′ CTCCTGTCTTGCTGCCATGG 3′	ChIP-qPCR	Sangon Biotech, Shanghai, China
P2-5-F	5′ AACCAGCACCTCCTCTTTCTTGTTC3′	ChIP-qPCR	Sangon Biotech, Shanghai, China
P2-5-R	5′ CCCTCCAAACCTGCCTATTGTGTTAG 3′	ChIP-qPCR	Sangon Biotech, Shanghai, China
GSTA2-F	5′ TACTCCAATATACGGGGCAGAA 3′	qRT-PCR	Sangon Biotech, Shanghai, China
GSTA2-R	5′ TCCTCAGGTTGACTAAAGGGC 3′	qRT-PCR	Sangon Biotech, Shanghai, China
GCS-F	5′ GGAAGTGGATGTGGACACCAGATG 3′	qRT-PCR	Sangon Biotech, Shanghai, China
GCS-R	5′ ACACTGTCTTGCTTGTAGTCAGGATG 3′	qRT-PCR	Sangon Biotech, Shanghai, China
HO-1-F	5′ CCACCAAGTTCAAGCAGCTCTACC 3′	qRT-PCR	Sangon Biotech, Shanghai, China
HO-1-R	5′ ATGTTGAGCAGGAACGCAGTCTTG 3′	qRT-PCR	Sangon Biotech, Shanghai, China
NQO-1-F	5′ AAGCCGCAGACCTTGTGATATTCC 3′	qRT-PCR	Sangon Biotech, Shanghai, China
NQO-1-R	5′ CTCTCCTATGAACACTCGCTCAAACC3′	qRT-PCR	Sangon Biotech, Shanghai, China

### Western Blotting

Cells were lysed with RIPA (Beyotime, Beijing, China) on ice for 30 min, and then, cell lysates were centrifuged at the highest speed; the protein was in the supernatant. Next, protein concentration was analyzed using a BCA Protein Assay Kit (Beyotime, Beijing, China). Forty micrograms of each protein sample was separated via SDS-PAGE and transferred onto polyvinylidene fluoride (PVDF) membranes (GE Healthcare, United Kingdom). Next, the PVDF membranes were incubated with 5% BSA, followed by incubations with primary and secondary antibodies. Finally, the protein bands were visualized with GeneSnap using a SynGene system (Shanghai, China).

### Transfection of Cell Lines

All transfections were carried out in Opti-MEM (Gibco, Brooklyn, NY, United States) using Lipofectamine 3000 (Gibco, Brooklyn, NY, United States). After 48 h, cells were harvested for subsequent analysis. Lentiviruses were used to transduce cells according to the manufacturer’s instructions. Plasmids were obtained from Sangon (Shanghai, China). siRNAs were from RIBOBIO (Guangzhou, China). Lentiviruses were from GenePharma (Shanghai, China) and GeneChem (Shanghai, China).

### Cell Counting Kit 8 Assays

Cells were harvested after transfection with plasmids or siRNAs. Then, the cells were washed with PBS, resuspended in DMEM and plated into 96-well plates at a concentration of 3000 cells/well. Next, CCK-8 reagent (MCE, Shanghai, China) was added to the wells according to the manufacturer’s instructions. Optical density at 450 nm was measured using a microplate reader (Bio-Rad, Hercules, CA, United States) to assess cell viability.

### Colony-Formation Assays

Cells were harvested after transfection with plasmids or siRNAs. Then, the cells were washed with PBS, resuspended in DMEM and plated into six-well plates at a concentration of 500 cells/well. After 10 days, colonies were immobilized on the plates with 4% triformol for 20 min, stained with 0.5% crystal violet for 30 min and counted using ImageJ software.

### Cell Migration and Invasion Assays

Migration assays were performed to analyze cellular migration and invasion abilities. First, cells were harvested after transfection with plasmids or siRNAs. Then, the cells were washed with PBS and resuspended in DMEM without fetal bovine serum. Medium with 10% fetal bovine serum was added into the lower chambers, followed by the addition of 200 μL serum-free DMEM containing 5 × 10^4^ cells into the upper chambers. For invasion assays, matrigel matrix (Corning, Toledo, OH, United States) was injected into the chambers before cells addition. After 24–48 h, the migrating and invading cells were immobilized on chambers with 4% triformol for 20 min. Then, attached cells were stained with 0.5% crystal violet for 30 min, and the cell migration and invasion ability were evaluated through digital imaging of the cells.

### Dual-Luciferase Reporter Assays

Cells were seeded in 24-well plates at an appropriate concentration on the first day. Cells were transfected with plasmids, Renilla luciferase vector and siRNAs at an appropriate ratio on the second day. After 48 h, cells were washed with PBS and lysed in passive lysis buffer. The activities of firefly and Renilla luciferases were measured by using a Dual Luciferase Reporter Assay System (Promega, Madison, WI, United States) according to the manufacturer’s protocol. The luminescence intensities of firefly and Renilla luciferases were recorded using a microplate reader. The results are presented as relative firefly luciferase activity after normalization to the internal control Renilla luciferase activity. All results were obtained from at least three independent experiments.

### DNA Pull-Down Assay

The NRF2 promoter region located at 3000 bp upstream of the transcription initiation sites was amplified via PCR using a 5-biotin-labeled forward primer. The primer sequences were as follows: forward primer, reverse primer. The 5-biotinylated DNA of the 5-flanking region of the NRF2 promoter was immobilized to streptavidin beads following the manufacturer’s protocol (Dynabeads^®^ kilobase BINDERTM kit, Invitrogen Dynal AS, Oslo, Norway). Proteins in the nuclear fraction were incubated with 5-biotinylated DNA beads on a rotating shaker at 4°C overnight. Following this incubation, the supernatant was removed. The beads were washed three times with cold PBS. After the last wash, the pull-down mixture was resuspended in distilled water at 70°C for 3 min to break the bond between streptavidin and biotin. The proteins eluted from the beads were subjected to WB and MS analyses. The proteins eluted from the beads without the biotinylated DNA probe were used as a control.

### Co-immunoprecipitation (Co-IP)

Cells were harvested, and the proteins were extracted, quantified as previously described and then incubated with antibodies and protein A/G beads for IP testing using a co-immunoprecipitation (Co-IP) kit (Active Motif, Carlsbad, CA, United States) according to the manufacturer’s instructions. Immunoprecipitated proteins were detected via western blotting. The antibodies used are listed in Table 2.

**TABLE 2:** Antibodies used in this paper.

Antibodies	Catalog number	Application	Company
Anti-Telomerase reverse transcriptase	ab32020	WB, IP,I F, IHC	Abcam, Cambridge, MA, United States
NRF2 (D1Z9C) XP^®^ Rabbit mAb	12721S	WB, ChIP	Cell Signaling Technology, United States
Anti-Nrf2[EP1808Y]	ab62352	WB, IHC	Abcam, Cambridge, MA, United States
YBX1 Polyclonal Antibody	20339-1-AP	IHC, WB	ProteinTech, Wuhan, China
Anti-YB1 antibody [4F12]	ab219070	IF	Abcam, Cambridge, MA, United States
Anti-GAPDH [EPR16891]	Ab181602	WB	Abcam, Cambridge, MA, United States

### Chromatin Immunoprecipitation (ChIP)

Chromatin immunoprecipitation (ChIP) assays were performed according to the manufacturer’s protocol (CST, Boston, MA, United States). The percentage of bound DNA was quantified against the original DNA input. Specific primers were designed to amplify the NRF2 promoter sequence, which was immunoprecipitated with a specific anti-YB1 antibody. The primers used for amplification of the precipitated DNA fragments are listed in Table 1. PCR products were analyzed via agarose gel electrophoresis.

### Immunofluorescence Staining (IF)

Cells were grown on a circular microscope cover glass until 30–40% confluency was achieved. For cell fixation, cells were incubated with 4% paraformaldehyde for 20 min at room temperature. Subsequently, cells were washed twice with PBS, followed by permeabilization with 0.2% Triton-X/PBS for 15 min and blocking with 1% (w/v) BSA at room temperature for 30 min. Primary antibodies (1:250) were diluted in 5% BSA (w/v) and incubated with cells at 4°C overnight. Washing was performed thrice with PBS. Next, cells were incubated with goat anti-rabbit IgG CY3 (Beyotime, Beijing, China) and goat anti-mouse IgG FITC secondary antibody (Beyotime, Beijing, China) at room temperature in the dark for 1 h. The cover glass was removed following three additional washes with PBS and mounted on a coverslip with PBS containing DAPI (Beyotime, Beijing, China), which counterstained the nuclei. Subsequently, a confocal microscope (Olympus, Japan) was used to visualize the stained cells at 600× magnification.

### Statistical Analysis

Statistical analyses were conducted using SPSS 22.0 software (IBM, United States). All data are presented as the means ± SD. A *t*-test was performed to compare variables between two groups, and ANOVA was used for multigroup comparisons. Correlations between the expression levels of hTERT, YBX1, and NRF2 were analyzed using a Spearman test. Differences with *p* < 0.05 were considered statistically significant.

## Results

### hTERT and NRF2 Are Highly Expressed in CRC Tissues and Associated With Poor Diagnosis

**FIGURE 1:**
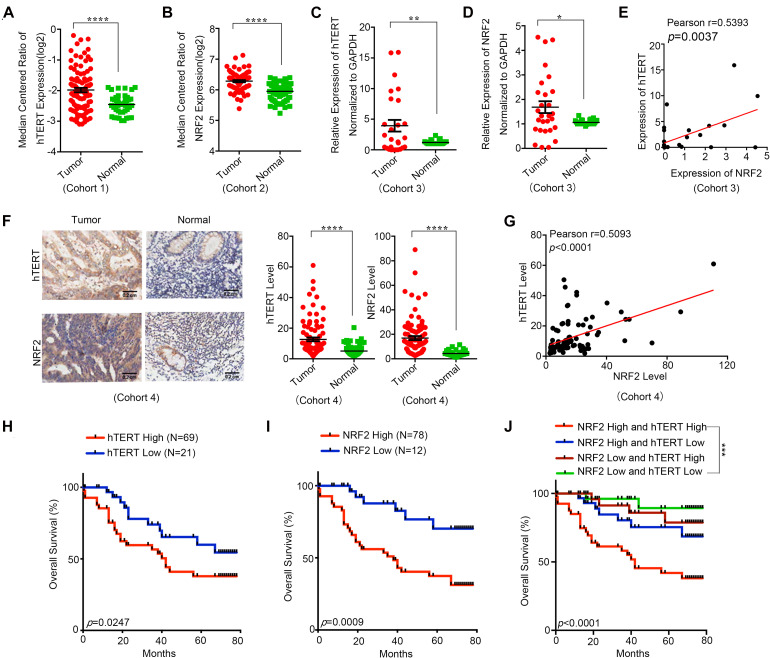
Human telomerase reverse transcriptase (hTERT) and NRF2 are highly expressed in CRC tissues and associated with poor diagnosis. **(A)** hTERT expression in CRC tissues and adjacent normal tissues was investigated in Oncomine database (Cohort1). **(B)** NRF2 expression in CRC tissues and adjacent normal tissues was investigated in Oncomine database (Cohort2). **(C)** hTERT mRNA expression in CRC tissues and paired adjacent normal tissues was identified by qRT-PCR (Cohort3). **(D)** NRF2 mRNA expression in CRC tissues and paired adjacent normal tissues was identified by qRT-PCR (Cohort3). **(E)** Regulation analysis of the correlation between hTERT and NRF2 (Cohort3). Each point represents one cancer sample. **(F)** Representative immunohistochemical staining and expression level statistics of hTERT and NRF2 in CRC tissues and paired adjacent normal tissues (Cohort4). **(G)** Regulation analysis of the correlation between hTERT and NRF2 (Cohort4). Each point represents one cancer sample. **(H)** Kaplan–Meier analysis of the overall survival of CRC patients with different hTERT expression levels (*p* < 0.05, log-rank test). **(I)** Kaplan–Meier analysis of the overall survival of CRC patients with different NRF2 expression levels (*p* < 0.05, log-rank test). **(J)** Kaplan–Meier analysis of the overall survival of CRC patients with different hTERT and NRF2 expression levels (*p* < 0.05, log-rank test). *p < 0.05; **p < 0.01; ***p < 0.001; ****p < 0.0001; ns, no significance.

In this study, we first explored the hTERT and NRF2 expression levels in CRC tumor tissues and adjacent normal tissues and their effect on CRC patients. According to the Oncomine database, both hTERT and NRF2 were more highly expressed in CRC tumor tissues than in adjacent normal tissues (Figures 1A,B). In addition, our collected samples also showed that hTERT and NRF2 mRNA levels were higher in tumor tissues than in paired adjacent normal tissues (Figures 1C,D). Meanwhile, we found that hTERT mRNA was positively correlated with NRF2 mRNA in CRC tissues (Figure 1E). Furthermore, we identified the hTERT and NRF2 expression levels in another cohort containing 90 CRC tissues and paired adjacent normal tissues. Similarly, immunohistochemical (IHC) staining revealed that both hTERT and NRF2 showed a higher level in CRC tissues than in the adjacent normal tissues. In the 90 pairs of CRC tissues, the expression of both hTERT and NRF2 was higher than in adjacent normal tissues (Figure 1F), and hTERT was positively correlated with NRF2 expression (Figure 1G). Overall survival curve analysis indicated that CRC patients with high hTERT or NRF2 expression had relatively poorer overall survival than those with low hTERT or NRF2 expression after surgery (Figures 1H,I). Moreover, CRC patients with high hTERT expression and concomitantly high NRF2 expression had the worst survival after surgery (Figure 1J). Meta-analysis showed that among the eight variables (tumor size, vascular invasion, clinical stage, age, sex, distal metastasis, pathology grade, and lymphatic metastasis), hTERT expression, vascular invasion, clinical stage, lymphatic metastasis, pathology grade and sex, were independent risk factors for CRC survival (Supplementary Figure 1A), while NRF2 expression, age, sex, distal metastasis, vascular invasion, lymphatic metastasis, and clinical stage were independent risk factors for CRC survival (Supplementary Figure 1B). ROC curves illustrated that the area under the curve (AUC) of the hTERT- and NRF2-based predictions were 0.7679 and 0.86878, respectively, suggesting that both could potentially be applied for prediction of patient survival (Supplementary Figures 1C,D).

Collectively, these results indicate that both hTERT and NRF2 are highly expressed in CRC patients, and their expression has a positive relationship, suggesting a potential hTERT/NRF2 pathway in CRC tissues. Moreover, a higher hTERT or NRF2 expression level was associated with a shorter survival time after surgery, and patients with both high hTERT expression and high NRF2 expression showed the worst survival, indicating that both hTERT and NRF2 have an important role in CRC and could be applied for predicting the prognosis of CRC patients.

### hTERT Upregulates NRF2 Expression by Promoting NRF2 Transcriptional Activity

**FIGURE 2:**
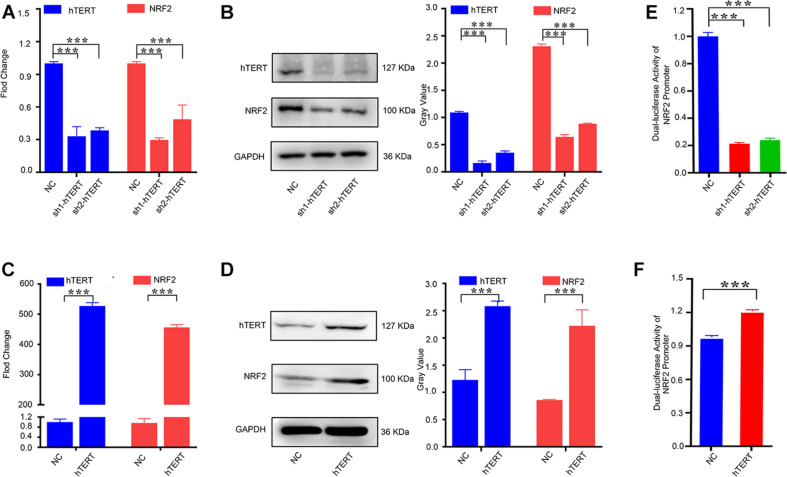
Human telomerase reverse transcriptase (hTERT) upregulates NRF2 expression by promoting NRF2 transcriptional activity. **(A)** hTERT-knockdown cells were constructed by transfecting sh1-hTERT and sh2-hTERT lenviral respectively into HCT 116 cells. The hTERT and NRF2 mRNA expression level after downregulation of hTERT were identified by qRT-PCR. **(B)** hTERT and NRF2 protein expression levels after downregulation of hTERT were detected by western blotting (Left). Statistical analysis of western blotting (Right). **(C)** hTERT-overexpressed cell was constructed by transfecting hTERT lenviral into SW620 cells. The hTERT and NRF2 mRNA expression level after upregulation of hTERT was identified by qRT-PCR. **(D)** hTERT and NRF2 protein expression levels after overexpression of hTERT were detected by western blotting (Left). Statistical analysis of western blotting (Right). **(E)** Luciferase activity of the NRF2 promoter was detected after downregulation of hTERT. **(F)** Luciferase activity of NRF2 was detected after overexpression of hTERT. *p < 0.05; **p < 0.01; ***p < 0.001; ns, no significance.

Because a positive correlation was found between hTERT and NRF2 expression in CRC tissues, we investigated whether hTERT could regulate NRF2 expression in CRC cells. Before that, we first examined the expression of hTERT and NRF2 in a variety of human CRC cell lines via qPCR. hTERT expression was low in SW460, SW480, and SW620 cells; moderate in HT29 and LOVO cells; and high in HCT116 and RKO cells (Supplementary Figure 1E). NRF2 expression was low in SW620, SW480, and SW620 cells; moderate in HT29, LOVO, and RKO cells; and high in HCT116 cells (Supplementary Figure 1F). We selected HCT116 cells as the model for stable downregulation of hTERT or NRF2 and SW620 cells as the model for stable hTERT overexpression. We next performed qRT-PCR analysis, which showed that knockdown of hTERT decreased the NRF2 mRNA expression level (Figure 2A). Western blotting showed similar results: hTERT knockdown decreased NRF2 expression (Figure 2B). However, overexpression of hTERT increased NRF2 mRNA expression (Figure 2C), and western blotting showed similar results at the protein level, with hTERT overexpression accelerating NRF2 expression (Figure 2D). As previously reported, NRF2 is negatively regulated by KEAP1. Under normal physiological conditions, NRF2 in the cytoplasm is degraded by cullin 3 (CUL3), which is a protein in the KEAP1 complex (Sporn and Liby, 2012). When exposed to ROS, NRF2 dissociates from KEAP1 and translocates to the nucleus (Ge et al., 2017). NRF2 can also be activated through the autophagy-lysosome pathway (Fan et al., 2010). Therefore, how is NRF2 regulated by hTERT? Our research revealed that knockdown of hTERT did not reduce the decay of the NRF2 mRNA level induced by actinomycin D treatment (Supplementary Figure 3A). In addition, overexpression of hTERT did not increase the stability of the NRF2 protein after CHX treatment (Supplementary Figure 3B). However, promoter luciferase assays indicated that knockdown of hTERT decreased NRF2 promoter activity (Figure 2E), while overexpression of hTERT increased NRF2 promoter activity (Figure 2F). The above results suggest that hTERT upregulates NRF2 not by increasing NRF2 mRNA or protein stability but by promoting NRF2 promoter activity, thus increasing NRF2 transcription.

### hTERT Promotes Colorectal Cancer Proliferation and Migration by Upregulating NRF2

**FIGURE 3:**
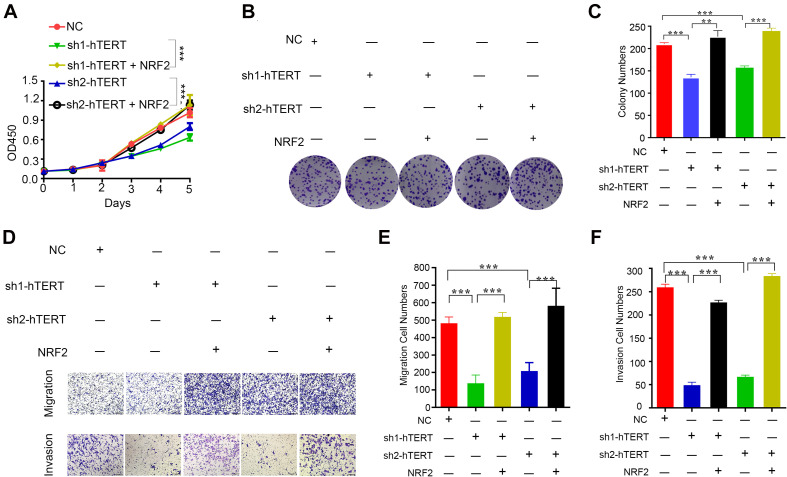
Human telomerase reverse transcriptase (hTERT) promotes colorectal cancer cell proliferation and metastasis by upregulating NRF2. **(A)** CCK8 assays were performed to detect cell proliferation after downregulation of hTERT but an increase in NRF2 expression. **(B)** Colony formation assays were performed after downregulation of hTERT but an increase in NRF2 expression. **(C)** Statistical analysis of the colony numbers. **(D)** Migration and invasion assays were performed after downregulation of hTERT but an increase in NRF2 expression. **(E)** Statistical analysis of the migration cell numbers. **(F)** Statistical analysis of the invasion cell numbers. *p < 0.05; **p < 0.01; ***p < 0.001; ns, no significance.

Because hTERT and NRF2 have previously been reported to promote CRC progression (Minafra et al., 2017; Sadeghi et al., 2018), we first verified the function of hTERT and NRF2 using CCK8, colony formation and transwell assays. The results showed that knockdown of hTERT expression in HCT116 cells significantly decreased cell viability (Supplementary Figure 2A), colony formation capacity (Supplementary Figure 2B), cell migration and invasion (Supplementary Figures 2C–E). Similarly, knockdown of NRF2 expression in HCT116 cells significantly decreased cell proliferation (Supplementary Figures 2F,G), migration and invasion ability (Supplementary Figures 2H–J). Interestingly, we then performed rescue experiments with two hTERT-knockdown strains, which revealed that overexpression of NRF2 could rescue the hTERT-knockdown-mediated suppression of cell proliferation (Figures 3A–C), migration and invasion (Figures 3D–F). We also performed cell cycle arrest and apoptosis analysis. However, there were no difference in cell cycle process with hTERT knockdown and NRF2 overexpression (Supplementary Figure 2K). hTERT decrease could promote CRC apoptosis, while enhancing NRF2 expression could rescue the apoptosis (Supplementary Figure 2L).

From these results, we conclude that both hTERT and NRF2 can increase CRC cell proliferation, colony formation and migration and that hTERT promotes CRC proliferation and migration by relying on upregulation of NRF2 at the transcriptional level.

### hTERT Increases NRF2 Expression by Recruiting YBX1 to Bind to the NRF2 Promoter

**FIGURE 4:**
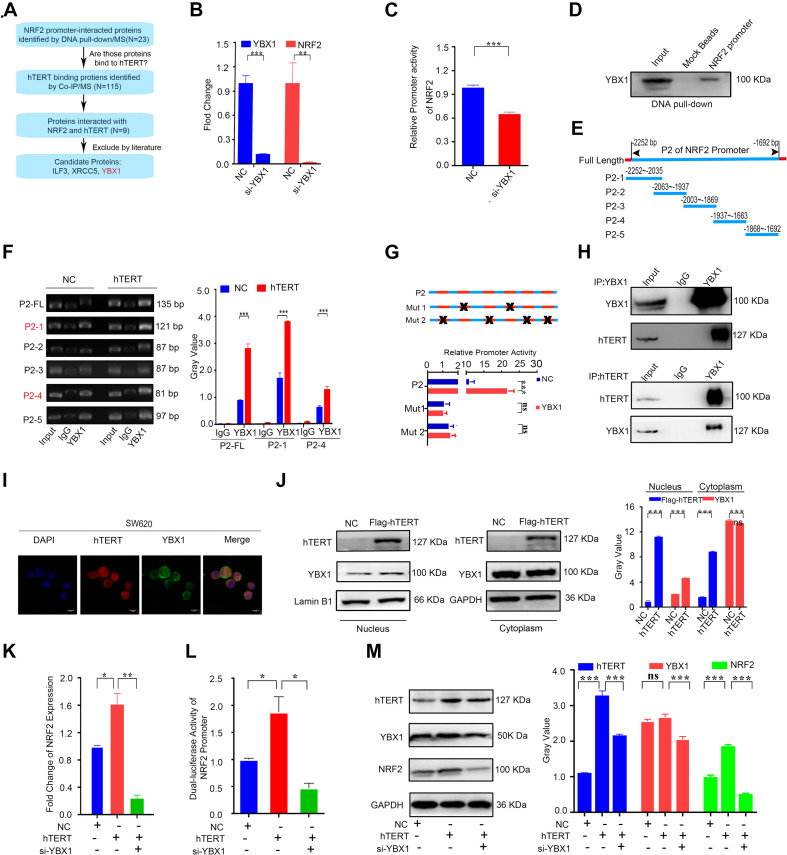
Human telomerase reverse transcriptase (hTERT) increases NRF2 expression by recruiting YBX1 to bind to the NRF2 promoter. **(A)** Flow chart for screening of potential hTERT-recruited NRF2 transcription factors. **(B)** The YBX1 and NRF2 mRNA expression level was identified by qRT-PCR after downregulation of YBX1. **(C)** Luciferase activity of the NRF2 promoter was detected after downregulation of YBX1. **(D)** NRF2 promoter with a 5′ biotin label was used to pull-down YBX1. Mock beads were used as a negative control. **(E)** Diagrammatic drawing of NRF2 P2 fragment. The P2 fragment was divided into 5 fragments. **(F)** YBX1 antibody was used to immunoprecipitate binding fragments of the NRF2 promoter under the condition of hTERT overexpression and fragments were identified by ChIP-qPCR with six primers (Left). Statistical analysis of ChIP-qPCR. **(G)** Luciferase activity of P2 fragment containing different mutant sites was detected after YBX1 overexpression. **(H)** HCT116 cell lysates were prepared for separate IP with hTERT and YBX1 antibody and then evaluated via western blotting. **(I)** The subcellular localization and the colocalization of hTERT and YBX1 were examined in SW620 cells via dual immunofluorescence using confocal microscopy. **(J)** YBX1 in cell nuclei and cytoplasm was identified by western blotting after overexpression of Flag-hTERT. **(K)** The NRF2 mRNA expression level was identified by qRT-PCR after overexpression of hTERT and simultaneous knockdown of YBX1. **(L)** Luciferase activity of the NRF2 promoter was detected after overexpression of hTERT and simultaneous knockdown of YBX1. **(M)** The NRF2 protein level was identified by western blotting after overexpression of hTERT and simultaneous knockdown of YBX1 (Left). Statistical analysis of western blotting (Right). *p < 0.05; **p < 0.01; ***p < 0.001; ns, no significance.

Our previous study demonstrated that hTERT could promote NRF2 expression at the mRNA level. Although hTERT is not a transcription factor, it has been reported to regulate gene expression by interacting with specific transcriptional factors that bind to the promoters of their target genes, thus regulating gene transcription (Hu et al., 2017). To identify the potential transcription factor that interacts with NRF2, we first amplified the 5′ biotin-labeled NRF2 promoter region located 3000 bp upstream of the *NRF2* gene, and the 5′ biotin-labeled NRF2 promoter was subsequently used to pull-down its associated transcription factors. hTERT immunoprecipitation (IP) followed by LC-MS was performed to identify proteins that could potentially bind with hTERT. Both the pull-down and IP were performed three times. In total, 23 proteins were found to bind to the NRF2 promoter, and 115 proteins were identified as hTERT binding proteins. Nine of those proteins were intersected between the two sets. Of those nine proteins, we screened ILF3, XRCC5, and YBX1 as candidate transcription factors that could bind to hTERT (Figure 4A). ILF3 has been reported as a transcription factor that promotes cell proliferation over differentiation in K562 erythroleukemia cells (Wu et al., 2018). ILF3 is also highly expressed in CRC patients and correlated with a poor CRC prognosis (Li et al., 2019). XRCC5 knockdown suppressed binding of XRCC5 to the CLC-3 promoter and decreased its promoter activity in gastric cancer (Gu et al., 2018). XRCC5 also acts as a binding factor by cooperating with P300 to bind to the COX-2 gene promoter, thus increasing COX-2 expression and subsequently promoting colon cancer growth (Zhang Z. et al., 2017). YBX1 was reported as a great potential therapeutic target and prognostic biomarker for CRC (Yan et al., 2014). YBX1 is also a highly conserved transcription factor. Liu Z. et al. (2020) reported that YBX1 binds to the GSK3B promoter to promote pancreatic cancer growth.

To further screen the NRF2 transcription factor(s), we first performed separate knockdown of ILF3, XRCC5, and YBX1 using siRNA and subsequently conducted qPCR and dual luciferase reporter assays. The results showed that YBX1 inhibition significantly decreased NRF2 mRNA expression (Figure 4B) and NRF2 promoter activity (Figure 4C), while knockdown of ILF3 only decreased NRF2 mRNA expression by 20% compared to the control group (Supplementary Figure 3C) and knockdown of ILF3 did not downregulate NRF2 promoter activity (Supplementary Figure 3D). Inhibition of XRCC5 did not decrease NRF2 mRNA expression (Supplementary Figure 3C) or NRF2 promoter activity (Supplementary Figure 3D). In sum, YBX1 was identified as the potential transcription factor that interacts with NRF2. Furthermore, the abovementioned 5′ biotin-labeled NRF2 promoter was used to pull down its interacting proteins, which verified that YBX1 could bind to the NRF2 promoter (Figure 4D). Meanwhile, we performed a ChIP assay followed by qPCR to examine which area of the NRF2 promoter was the binding region for YBX1. Before performing ChIP, we predicted the YBX1 binding sites on the NRF2 promoter using JASPER software. According to the predicted binding sites, we designed five pairs of primers that spanned the sequence from +1 to −3000 bp of the NRF2 promoter. We named the corresponding five fragments P1 to P5. Each fragment had several JASPER software-predicted binding sites in the NRF2 promoter. The detailed fragments of the NRF2 promoter are shown in Supplementary Figure 3F. From the YBX1 ChIP-qPCR results, only the P2 region could be amplified, which indicated that YBX1 specifically binds to the P2 region of the NRF2 promoter (Supplementary Figure 3G). There were six predicted YBX1 binding sites on the P2 fragment: two of the binding sequences contained “CCAAT,” which is believed to be the YBX1 DNA binding site (Chen et al., 2004), and the other four predicted YBX1 binding sites have not been reported. In order to identify the exact YBX1 binding sites at P2 fragment, we divided the P2 region into five fragments (P2-1 to P2-5) according to the six predicted binding sites, and five primers were designed to perform ChIP assay once more. The target fragments of each primer were illustrated in Figure 4E. Meanwhile, to confirm the role of hTERT in enhancing the YBX1-NRF2 promoter binding, ChIP experiment was proceeding under the condition of hTERT overexpression. Our results showed that with hTERT overexpression, increased P2 fragments could bind with YBX1, especially fragment P2-1 and P2-4 (Figure 4F and Supplementary Figure 3H), suggesting that hTERT promotes YBX1 binding to NRF2 promoter, and the P2-1 and P2-4 were potential binding regions. For the above six binding sites on the P2 fragment, Three luciferase reporter plasmids containing different types of mutations were constructed: plasmid FL contained the full-length P2 fragment, FL with “CCAAT” deletion was named mut1, and FL with deletion of the four other binding sites was named mut2. Dual luciferase reporter assays for the three mutants showed that deletion of the binding sites on the P2 fragment significantly decreased P2 luciferase activity, and overexpression of YBX1 could not rescue the mutants-induced decrease in NRF2 promoter activity (Figure 4G). Further, knockdown of YBX1 with siRNA did not change the promoter activity with P2 mutation (Supplementary Figure 3J), suggesting that in addition to CCAAT, other binding sites for YBX1 likely exist on the NRF2 promoter, such as CCACC, CTTCCCA, TCCTC, or TCCTCC.

On the other hand, to confirm whether hTERT can recruit YBX1 in CRC cells, we performed hTERT and YBX1 immunoprecipitation, which showed that hTERT and YBX1 could immunoprecipitate each other (Figure 4H), revealing that hTERT and YBX1 formed a complex in CRC cells. Immunofluorescence (IF) showed that both hTERT and YBX1 are located in the nucleus of CRC cells (Figure 4I and Supplementary Figure 3I). Further, hTERT overexpression enhanced accumulation of YBX1 in the nucleus (Figure 4J), demonstrating that YBX1 promotes NRF2 transcriptional activity by relying on hTERT recruitment of YBX1 to translocate from the cytoplasm to the nucleus. Similar to previous results, qPCR and dual luciferase reporter assays showed that hTERT increased NRF2 mRNA expression and NRF2 promoter activity; however, knockdown of YBX1 decreased the hTERT-mediated increase in NRF2 mRNA (Figure 4K) and promoter activity (Figure 4L). Changes in the NRF2 protein level corresponded with changes in NRF2 mRNA (Figure 4M). Meanwhile, NRF2 target genes also overexpressed under the condition of hTERT or YBX1 overexpression (Supplementary Figure 3E). Based on the above results, we concluded that hTERT increases NRF2 expression to promote CRC progression by interacting with YBX1.

In sum, these results demonstrate that hTERT upregulates NRF2 by recruiting the transcription factor YBX1, which binds to the P2 fragment of the NRF2 promoter to increase the transcriptional activity of NRF2.

### YBX1 Is Responsible for Upregulation of NRF2 Expression and CRC Proliferation and Migration

**FIGURE 5:**
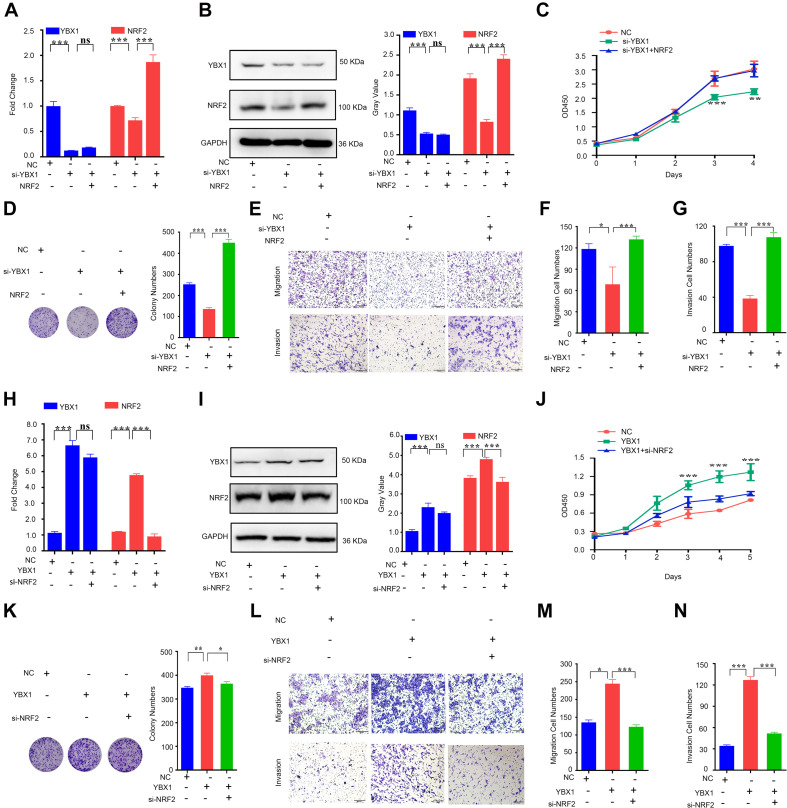
YBX1 is responsible for upregulation of NRF2 expression and CRC proliferation and migration. **(A)** The YBX1 and NRF2 mRNA expression level were identified by qRT-PCR after downregulation of YBX1 and simultaneous overexpression of NRF2. **(B)** The YBX1 and NRF2 protein expression level were identified via western blotting after downregulation of YBX1 and simultaneous overexpression of NRF2 (Left). Statistical analysis of western blotting (Right). **(C)** CCK8 assays were performed to detect cell proliferation after downregulation of YBX1 but an increase in NRF2 expression. **(D)** Colony formation assays were performed after downregulation of YBX1 but an increase in NRF2 expression (Left). Statistical analysis of the colony numbers (Right). **(E)** Migration and invasion assays were performed after downregulation of YBX1 but an increase in NRF2 expression. **(F)** Statistical analysis of the migration cell numbers. **(G)** Statistical analysis of the invasion cell numbers. **(H)** The YBX1 and NRF2 mRNA expression level were identified by qRT-PCR after overexpression of YBX1 and simultaneous downregulation of NRF2. **(I)** The YBX1 and NRF2 protein expression level were identified via western blotting after overexpression of YBX1 and simultaneous downregulation of NRF2 (Left). Statistical analysis of western blotting (Right). **(J)** CCK8 assays were performed to detect cell proliferation after overexpression of YBX1 and downregulation of NRF2. **(K)** Colony formation assays were performed after overexpression of YBX1 and downregulation of NRF2 (Left). Statistical analysis of the colony numbers (Right). **(L)** Migration and invasion assays were performed after overexpression of YBX1 and downregulation of NRF2. **(M)** Statistical analysis of the migration cell numbers. **(N)** Statistical analysis of the invasion cell numbers. *p < 0.05; **p < 0.01; ***p < 0.001; ns, no significance.

To further explore the effect of YBX1 on NRF2 and CRC progression, we again verified the effect of YBX1 on NRF2 expression. Our data showed that, similar to previous results, inhibition of YBX1 decreased the NRF2 mRNA level in CRC cells and that transfection with NRF2 plasmids rescued the NRF2 decrease caused by YBX1 knockdown (Figure 5A). The NRF2 protein level showed the same tendency as the NRF2 mRNA level (Figure 5B). Then, we identified the function of YBX1 and NRF2 in CRC cell proliferation, colony formation, migration and invasion. Knockdown of YBX1 was found to decrease CRC cell proliferation, colony conformation, migration and invasion, while increasing NRF2 expression via transfection with a NRF2 expression plasmid rescued CRC cell proliferation, colony conformation, migration and invasion (Figures 5C–G).

In contrast, overexpression of YBX1 induced by transfection with a YBX1 expression plasmid increased the NRF2 mRNA level, and transfection with NRF2 siRNA rescued the increase in NRF2 caused by YBX1 overexpression (Figure 5H). The NRF2 protein level showed the same tendency as the NRF2 mRNA level (Figure 5I). A function study showed that CRC cell proliferation, colony conformation, migration and invasion were promoted by YBX1 but attenuated by NRF2 inhibition (Figures 5J–N).

The above results suggest that YBX1 is responsible for upregulation of NRF2 at both the mRNA and protein levels and that YBX1 and NRF2 have the same function in promoting CRC cell proliferation and migration.

### YBX1 Is Highly Expressed in CRC and Associated With Poor Prognosis

**FIGURE 6:**
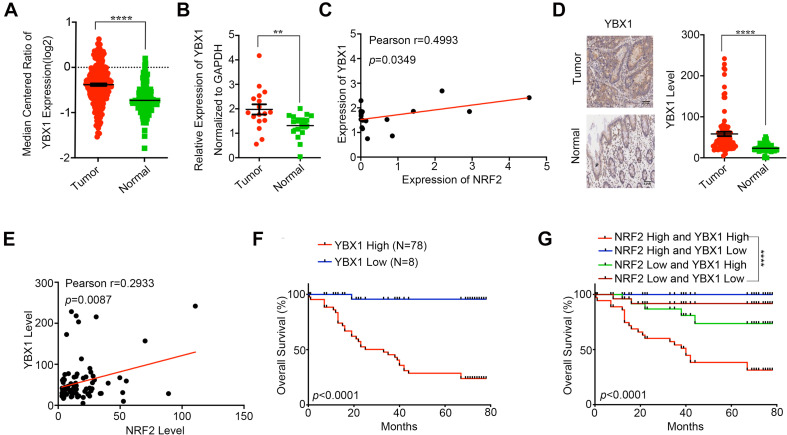
YBX1 is highly expressed in CRC and associated with poor prognosis. **(A)** YBX1 expression in CRC tissues and adjacent normal tissues was investigated in Oncomine database. **(B)** YBX1 mRNA expression in CRC tissues and paired adjacent normal tissues was identified by qRT-PCR. **(C)** Regulation analysis of the correlation between YBX1 and NRF2. Each point represents one cancer sample. **(D)** Representative immunohistochemical staining and expression level statistics of YBX1 in CRC tissues and paired adjacent normal tissues. **(E)** Regulation analysis of the correlation between YBX1 and NRF2. Each point represents one cancer sample. **(F)** Kaplan–Meier analysis of the overall survival of CRC patients with different YBX1 expression levels (*p* < 0.05, log-rank test). **(G)** Kaplan–Meier analysis of the overall survival of CRC patients with different YBX1 and NRF2 expression levels (*p* < 0.05, log-rank test). *p < 0.05;**p < 0.01; ***p < 0.001; ****p < 0.0001; ns, no significance.

YBX1 has been reported to be highly expressed in CRC tissue and to participate in CRC progression (Jürchott et al., 2010; Torres et al., 2017). Thus, we first investigated YBX1 in the Oncomine database and found that YBX1 showed higher expression in CRC tissues than in adjacent normal tissues (Figure 6A). We also identified YBX1 expression in CRC tissues via qPCR, which showed that YBX1 was expressed at higher levels in CRC tissues than in adjacent normal tissues (Figure 6B). Meanwhile, we found that YBX1 and NRF2 expression is positively correlated in CRC tissue (Figure 6C). Similarly, IHC staining revealed that YBX1 was highly expressed in CRC tissues compared with the adjacent non-cancer tissues. Of the 90 pairs of CRC tissues, the expression of YBX1 was higher in cancer tissues than in adjacent non-cancer tissues (Figure 6D), and YBX1 was positively expressed with NRF2 (Figure 6E). The overall survival curve analysis indicated that CRC patients with high YBX1 expression had a relatively poor prognosis compared with those with low YBX1 expression (Figure 6F). Moreover, CRC patients with high expression levels of both YBX1 and NRF2 had a significantly poorer prognosis (Figure 6G). Meta-analysis showed that YBX1 expression, together with the ten previously mentioned variables, was an independent risk factor of CRC patient survival (Supplementary Figure 3L). ROC curves illustrated that the AUC of the YBX1-based prediction was 0.8419, suggesting that YBX1 could also be applied for prediction of patient survival (Supplementary Figure 3K). The above data show that YBX1, acting as a transcription factor of NRF2, is highly expressed in CRC tissues and effects CRC prognosis along with NRF2.

## Discussion

**FIGURE 7:**
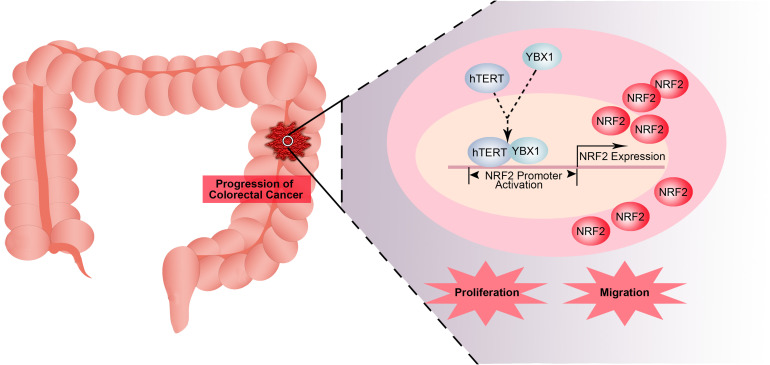
Model for transcriptional regulation of NRF2 by hTERT via recruitment of YBX1 in CRC proliferation and migration. In this model, hTERT recruits YBX1 to form a transcriptional complex, which binds to the NRF2 promoter to promote NRF2 expression, thus promoting CRC proliferation and migration.

In this study, we examined whether hTERT promotes CRC proliferation and migration by recruiting the transcription factor YBX1 to bind to the promoter of the NRF2 gene, activating NRF2 transcription (Figure 7). Our data demonstrated that hTERT, YBX1 and NRF2 are positively related in CRC tissues; explained why hTERT induces NRF2 upregulation; and found the binding sites on the NRF2 promoter that mediate its interaction with the transcription factor YBX1. Thus, we propose that hTERT promotes CRC progression through transcriptional regulation of NRF2 via recruitment of YBX1. Ultimately, this study provides a potential strategy and biomarkers for CRC prevention and treatment.

Human telomerase reverse transcriptase is the catalytic subunit of telomerase, which is located at the ends of chromosomes and protects them from degradation. It has been reported that hTERT and telomerase activity play a vital role in cell immortalization (Monsen et al., 2020), transformation (Yasukawa et al., 2020), proliferation (Wang et al., 2020), and metastasis (Hannen and Bartsch, 2018). A previous study indicated that hTERT could affect cancer development via activity not involving telomere elongation (Trybek et al., 2020). In the present study, our data provide strong evidence suggesting that hTERT plays a significant role in promoting CRC cell proliferation and migration. However, the mechanisms by which hTERT regulates cancer progression are diverse and complicated. Some studies have revealed that hTERT can regulate cancer progression by affecting ROS levels in cells. Indran et al. (2011) reported that hTERT overexpression not only reduced the basal cellular ROS levels but also inhibited endogenous ROS production in response to stimuli that induce intracellular ROS generation. Liu et al. (2016) demonstrated that hTERT decreased cellular ROS production, while maintaining mitochondrial function, and protected cells from hypoxia-induced apoptosis, which may attenuate the effects of irradiation-induced hypoxia in rectal injury following irradiation.

NFE2-related factor 2 is a molecule well known to regulate ROS homeostasis in cells. NRF2 is regulated by Keap1 via proteasomal degradation and is itself a transcription factor that activates target genes containing an ARE element (Shin et al., 2013; Khurana and Sikka, 2018). We found that both hTERT and NRF2 expression levels were upregulated in CRC tissues and that the expression level of hTERT was positively correlated with that of NRF2. High hTERT and NRF2 expression was found to be related to the shortest survival in CRC, suggesting that hTERT and NRF2 are potential prognostic markers and therapeutic molecular targets in CRC.

In our study, knockdown of hTERT markedly reduced NRF2 expression, while overexpression of hTERT significantly increased NRF2 expression in CRC cells at both the mRNA and protein level. Furthermore, knockdown of hTERT decreased NRF2 promoter activity, and overexpression of hTERT increased NRF2 promoter activity, suggesting that hTERT is closely associated with transcriptional regulation of NRF2 in CRC cells.

It is known that hTERT is not a transcription factor that directly regulates target gene transcription. hTERT might affect gene transcription by interacting with other transcription factors. It has been reported that hTERT can interact with BRG1 to regulate Wnt-dependent target genes (Park et al., 2009). Our previous study demonstrated that hTERT cooperated with c-Myc to upregulate heparanase expression (Tang et al., 2015). hTERT can also interact with ZEB1 to bind to the promoter of E-cadherin, subsequently promoting EMT in CRC (Qin et al., 2016). Based on these studies, we sought to investigate hTERT-interacting molecules that can function as transcription factors to promote transcriptional expression of NRF2. On the one hand, we searched for putative transcription factors that might bind to the NRF2 promoter region using a 5′ biotin-labeled NRF2 promoter to pull down the binding proteins. On the other hand, we used hTERT to immunoprecipitate the interacting proteins; “coincident” proteins from the two experiments were recognized as our target proteins. Different bioinformatics programs (PROMO, GeneCards, and JASPER) were subsequently used to screen the candidate transcription factors. We identified XRCC5, ILF3, and YBX1 for further analysis. Interestingly, we found that the hTERT-enhanced NRF2 expression and promoter activity were significantly decreased by knockdown of YBX1, suggesting that YBX1 might be associated with the hTERT-mediated transcriptional regulation of NRF2. Furthermore, ChIP analysis revealed that YBX1 bound to the promoter of NRF2. Moreover, YBX1-enhanced promoter activities were markedly abolished when the binding site of YBX1 was mutated, further demonstrating that hTERT-induced NRF2 promoter activity was YBX1-dependent.

YBX1 is a multifunctional molecule that can regulate DNA and RNA expression, impacting the progression of several cancer types (Lyabin et al., 2014). In our study, YBX1 was highly expressed in CRC and positively related to poor prognosis. YBX1-enhanced CRC proliferation, colony formation and migration were significantly decreased by knockdown of NRF2, while the YBX1-induced decrease in CRC progression was rescued by overexpression of YBX1, further suggesting that YBX1 acts upstream of NRF2. Our study found YBX1 not only binds to promoter sequences containing a “CCAAT” box but also likely binds to other sequences, such as “CCACC,” “CTTCCCA,” “TCCTC,” and “TCCTCCA” because mutation of these sites showed an ability to decrease the YBX1-induced NRF2 promoter activity similar to that of “CCAAT” box mutation. However, further detailed analysis should be performed to confirm the “true” binding sequences at the NRF2 promoter. Considering these data together, our study demonstrated that hTERT recruits YBX1 to bind to the NRF2 promoter and promotes NRF2 expression by increasing its transcriptional activity, thus ultimately increasing CRC progression. More, our study deeply analyzed the YBX1 binding sites and might have discovered novel binding sites on the NRF2 promoter.

In summary, our study shows that hTERT recruits the transcription factor YBX1 to bind to the NRF2 promoter, accelerating NRF2 transcriptional activity, increasing NRF2 expression, and thereby accelerating CRC proliferation and migration. This work may provide a new theoretical basis and a potential therapeutic target for prevention and treatment of CRC.

## Data Availability Statement

The original contributions presented in the study are included in the article/Supplementary Material, further inquiries can be directed to the corresponding author/s.

## Author Contributions

CG performed the experiments (cell culturing, DNA pull-down, ChIP-qPCR, Co-immunoprecipitation, colony formation assay, and migration and invasion assay), analyzed the data, interpreted the results, and drafted the manuscript. HY performed the experiments (Chromatin Immunoprecipitation and western blotting assay), analyzed the data, interpreted the results, and drafted the manuscript. SW and JL performed the IHC assay. ZL and YYH constructed the Plasmid vectors and analyzed the data. YC and YH performed the CCK8 assays. QL and YW performed the Immunofluorescence staining. EL conceived the study, interpreted the results, supervised the research, and wrote part of the manuscript. YX conceived the study, interpreted the results, and supervised the research. All authors read and approved the final manuscript.

## Conflict of Interest

The authors declare that the research was conducted in the absence of any commercial or financial relationships that could be construed as a potential conflict of interest.
