# Supplementary material for: hTERT Promotes CRC Proliferation and Migration by Recruiting YBX1 to Increase NRF2 Expression
Source: Front Cell Dev Biol. 2021 May 17;9:658101. doi: 10.3389/fcell.2021.658101 (PMC8165255; doi:10.3389/fcell.2021.658101)
Supplement: Supplementary file 1 [file Table_1.DOCX]

**Supplymentary legends**

Figure S1. hTERT and NRF2 are independent risk factors for CRC. **(A)** Meta-analyses of hazard ratios for overall survival using hTERT expression. **(B)** Meta-analyses of hazard ratios for overall survival using NRF2 expression. **(C)** Receiver operating characteristic (ROC) curves for predicting patient survival time using hTERT expression.**(D)** Receiver operating characteristic (ROC) curves for predicting patient survival time using NRF2 expression. **(E)** hTERT expression in CRC cell lines was detected by qRT-PCR. **(F)** NRF2 expression in CRC cell lines was detected by qRT-PCR.

Figure S2. hTERT or NRF2 promotes CRC cell proliferation, colony conformation and metastasis. **(A)** CCK8 assays were performed to detect cell proliferation after downregulation of hTERT. **(B)** Colony formation assays were performed after downregulation of hTERT (Left). Statistical analysis of the colony numbers (Right). **(C)** Migration and invasion assays were performed after downregulation of hTERT. **(D)** Statistical analysis of the migration cell numbers. **(E)** Statistical analysis of the invasion cell numbers. **(F)** CCK8 assays were performed to detect cell proliferation after downregulation of NRF2. **(G)** Colony formation assays were performed after downregulation of NRF2 (Left). Statistical analysis of the colony numbers (Right). **(H)** Migratoin and invasion assays were performed after downregulation of NRF2. **(I)** Statistical analysis of the migration cell numbers. **(J)** Statistical analysis of the invasion cell numbers. **(K)** Cell cycle assay after sh-hTERT and rescue by NRF2 overexpression**. (L)** Apoptosis assay after sh-hTERT and rescue by NRF2 overexpression.

Figure S3. YBX1, but not ILF3 or XRCC5, was found to be a hTERT-recruited transcription factor that mediated binding to the NRF2 promoter to increase NRF2 expression. **(A)** The NRF2 mRNA expression level in hTERT downregulation cells was identified by qRT-PCR after treatment with actinomycin D for different lengths of time. **(B)** The NRF2 protein expression level in hTERT overexpression cells was identified via western blotting after treatment with CHX for different lengths of time (Left). Statistical analysis of western bloting (Right). **(C)** The ILF3, XRCC5 and NRF2 mRNA expression level were identified by qRT-PCR after downregulation of ILF3 and XRCC5. **(D)** Luciferase activity of the NRF2 promoter was detected after downregulation of ILF3 and XRCC5. **(E)** The NRF2 target genes mRNA expression level were identified by qRT-PCR after hTERT and YBX1 overexpression. **(F)** Diagrammatic drawing of NRF2 promoter. The NRF2 promoter fragment was divided into 5 fragments. **(G)** YBX1 antibody was used to immunoprecipitate binding fragments of the NRF2 promoter, fragments were identified by ChIP-qPCR (Left). Statistical analysis of ChIP-qPCR (Right). **(H)** Statistical histogram of ChIP-qPCR. **(I)** The subcellular localization and the colocalization of hTERT and YBX1 were examined in HCT116 cells via dual immunofluorescence using confocal microscopy. **(J)** Luciferase activity of P2 fragment containing different mutant sites was detected after YBX1 knockdown. **(K)** Receiver operating characteristic (ROC) curves for predicting patient survival time using YBX1 expression. **(L)** Meta-analyses of hazard ratios for overall survival using YBX1 expression.
