# Supplementary figures and images for: hTERT Promotes CRC Proliferation and Migration by Recruiting YBX1 to Increase NRF2 Expression
Source: Front Cell Dev Biol. 2021 May 17;9:658101. doi: 10.3389/fcell.2021.658101 (PMC8165255; doi:10.3389/fcell.2021.658101)

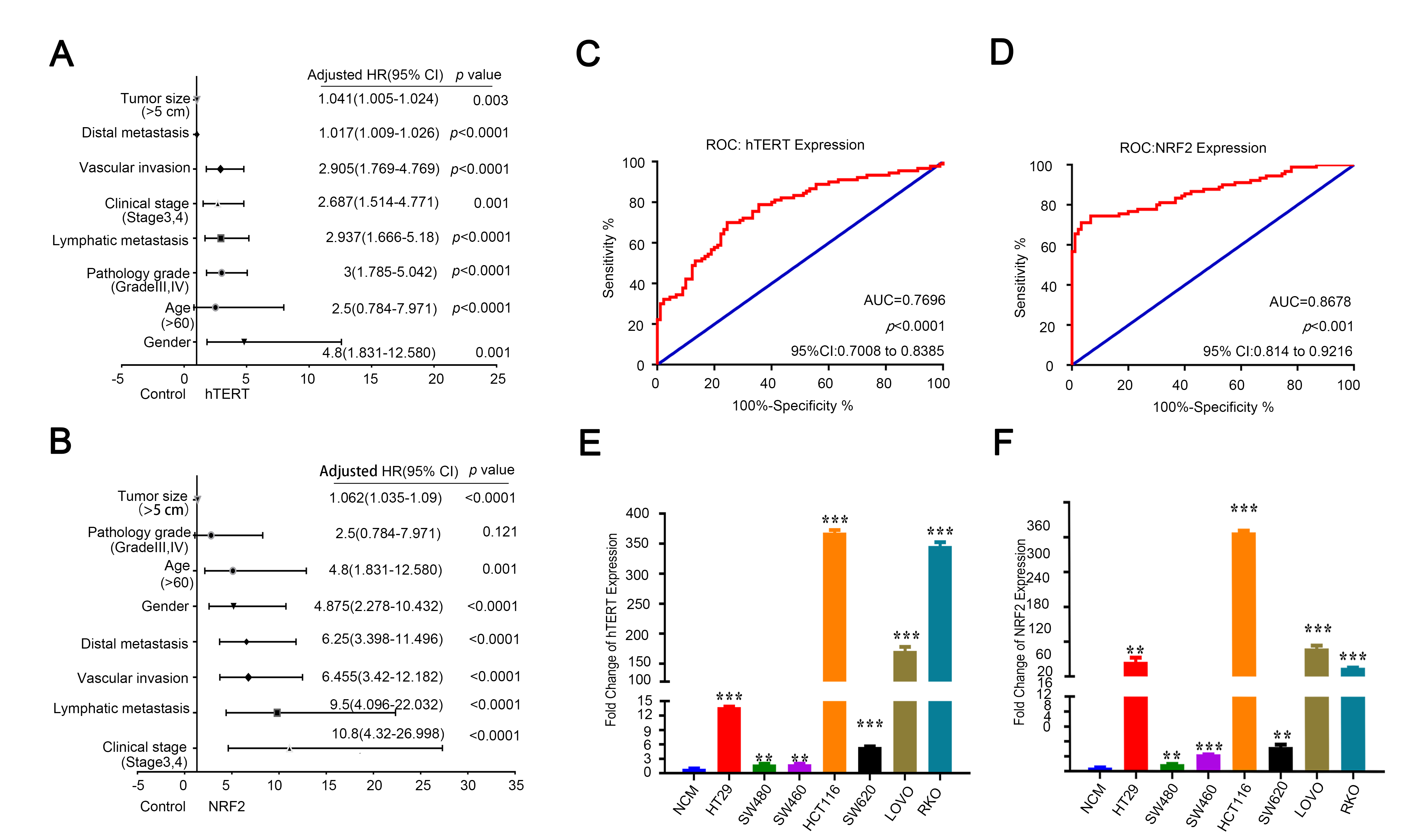

Supplement: Supplementary file 2 [file Image_1.TIF]

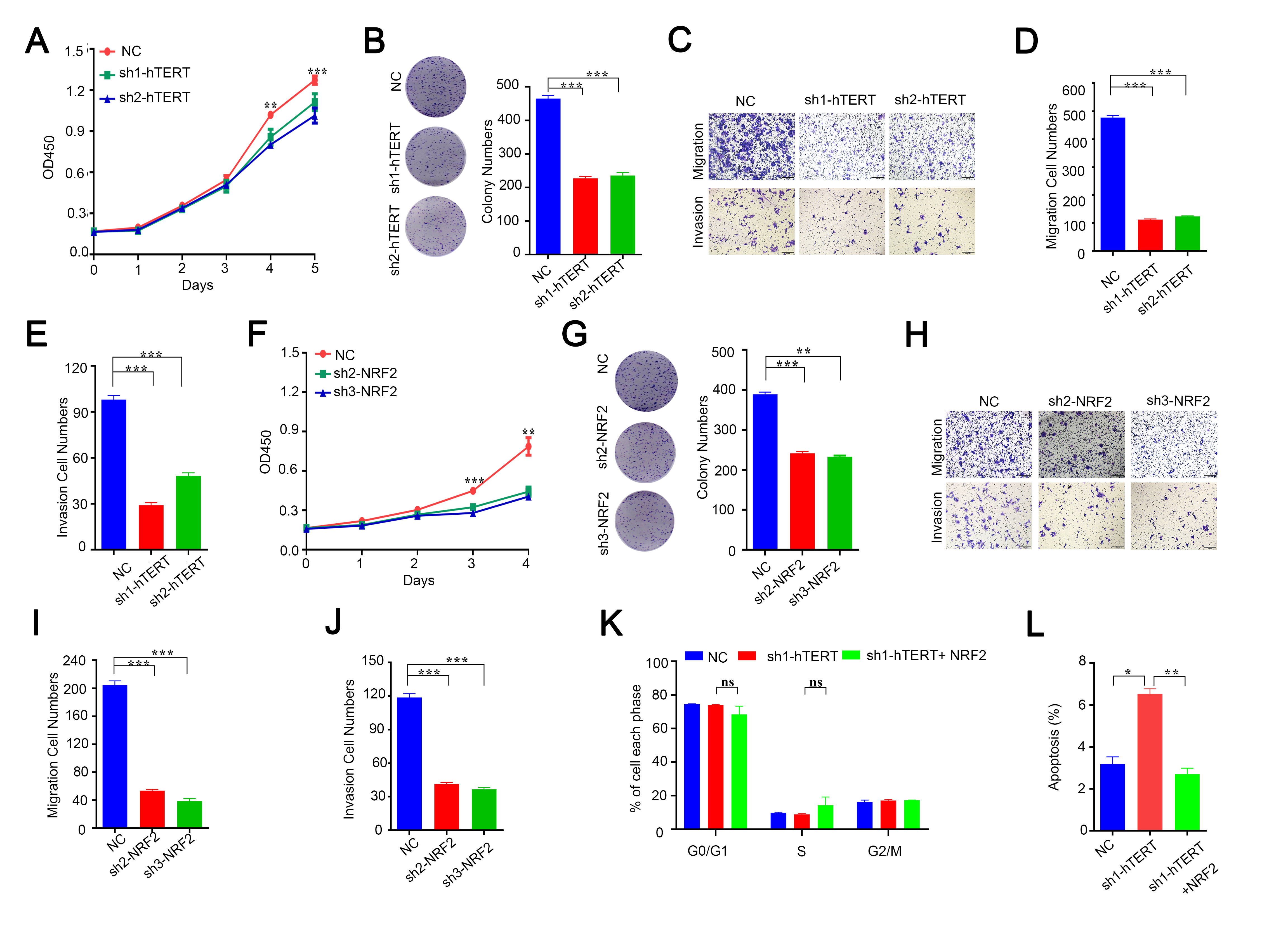

Supplement: Supplementary file 3 [file Image_2.TIF]

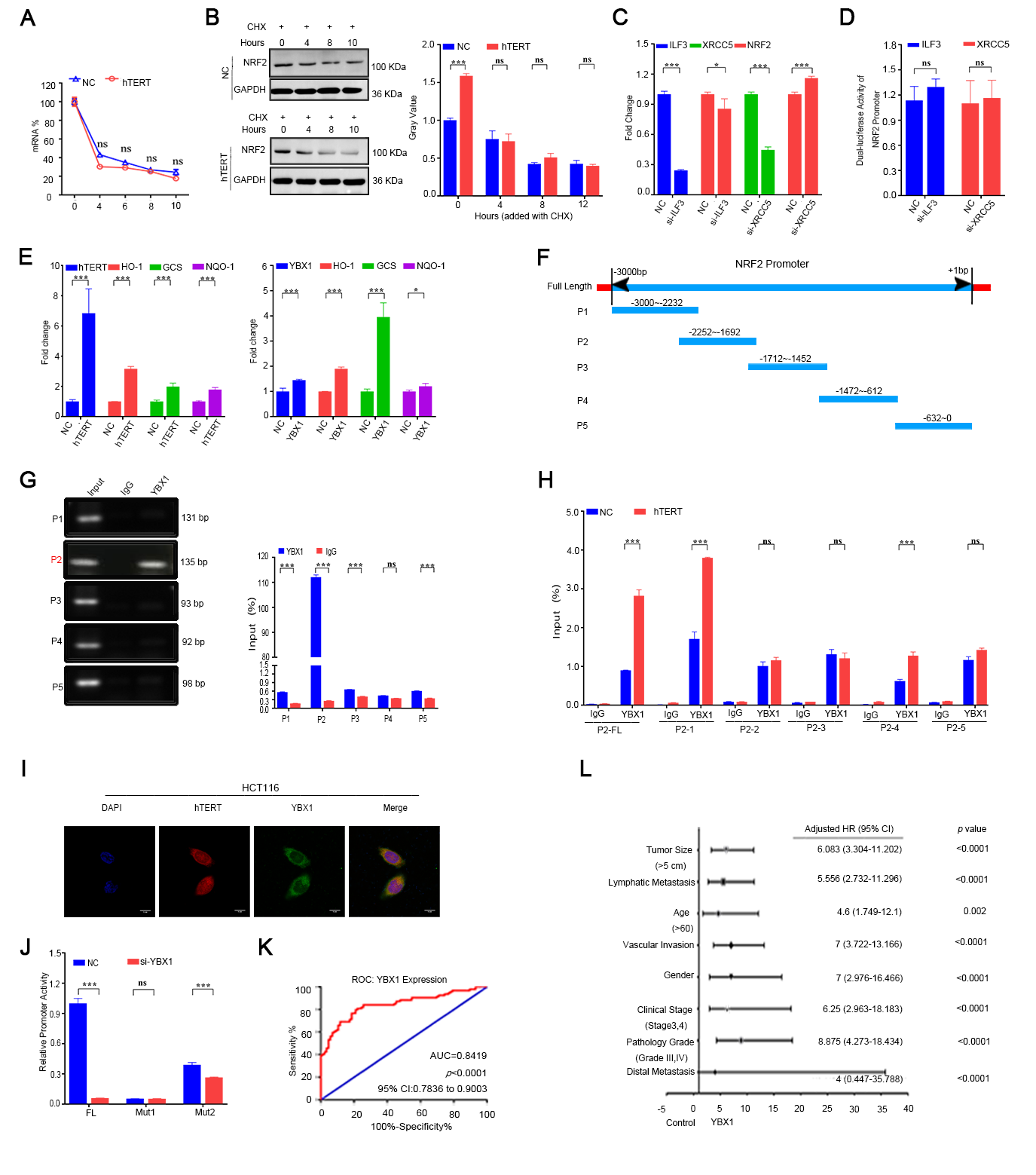

Supplement: Supplementary file 4 [file Image_3.TIF]
